# Career Preparedness and School Achievement of Portuguese Children: Longitudinal Trend Articulations

**DOI:** 10.3389/fpsyg.2017.00618

**Published:** 2017-04-24

**Authors:** Íris M. Oliveira, Maria do Céu Taveira, Erik J. Porfeli

**Affiliations:** ^1^Department of Applied Psychology, School of Psychology, University of MinhoBraga, Portugal; ^2^College of Medicine, Family and Community Medicine, Northeast Ohio Medical UniversityRootstown, OH, USA

**Keywords:** career preparedness, career development, school achievement, childhood, trend

## Abstract

Social Cognitive Career Theory suggests that students' preparedness for the school-to-work transition is a developmental process. Middle school children explore various careers, obtain feedback about their academic progress, and develop career self-efficacy and outcome expectations. These processes advance provisional educational/occupational goals. The literature has suggested articulations between career and academic development and how both vary across demographic characteristics, but longitudinal studies linking these processes are scarce. This study tested articulations between career preparedness and academic achievement during middle school years and employed gender and geographical location as potential moderators affecting the linkage between career and school domains. Participants included 429 children (47.8% girls) from northern (69.5%) and central Portugal (30.5%) followed across four occasions of measurement (*M*_ageWave1_ = 10.23, *SD* = 0.50). Data was collected with school records, the Multidimensional Scales of Perceived Self-Efficacy, Career Exploratory Outcome Expectations Scale, Childhood Career Exploration Inventory and Childhood Career Development Scale. Average and orthnormalized linear, quadratic and cubic trends were computed. Pearson correlation coefficients suggested positive and statistically significant associations between career exploratory outcome expectations and academic achievement average trends. Career planning and self-efficacy expectations were negatively associated with academic achievement quadratic trends. Multiple linear regression models suggested that career exploratory outcome expectations and career planning were respectively statistically significant predictors of the average and quadratic trends of academic achievement. Gender moderated the association between the career variables and academic achievement linear trends as well as the relation of career planning and self-efficacy with academic achievement cubic trends. Additionally, the geographical location moderated the association between the average trend of career exploratory outcome expectations and academic achievement as well as tended to moderate the relation between the career variables and academic achievement quadratic trends. Future research could seek to explore the role of context in shaping the trajectories and linkages between career and academic progress with a more representative sample of participants from a broader array of geographical locations. This study advances extant literature by affirming the longitudinal relationship between the school and work domains in youth, which might sustain practices aimed at fostering students' career preparedness and academic achievement.

## Introduction

Career development can be conceived as the sequence of life roles performed by an individual over his/her life-course (Super, 1994). The student role is of major importance in childhood (until the 14 years of age), as it sustains the development of work attitudes and routines as well as the awareness of one's abilities and preferences (Super, 1994; Araújo and Taveira, 2009). Their academic path promotes children's development of a sense of industry and identification with teachers who they may perceive as supportive and knowledgeable classroom managers who are concerned with their wellbeing (Erikson, 1963; Di Fabio and Kenny, 2015; Chávez, 2016; Longobardi et al., 2016). In addition, while performing the student role and being subject to its inherent social expectations and formal functioning (Super, 1980), children acquire literacy and numeric skills as well as identify social clues of one's gender, prestige, and capabilities, which are encapsulated in an emerging sense of self (Gottfredson, 1981).

Although career and academic development have been often separately considered, calls have been made to acknowledge their joint articulations (e.g., Watson et al., 2015). In consonance with such calls, theoretical perspectives articulating these processes have been advanced. Both career and academic literatures have moved from initial perspectives conceiving one's educational path and quality of teaching as the basis to acquire skills and sustain later occupational attainment (Dunkin and Biddle, 1974; Lombardi et al., 2011) to an integrative perspective assuming the complementarity among career and academic processes throughout the lifespan (Kuijpers and Meijers, 2012).

Aligned with such a complementary position, the Social Cognitive Career Theory (SCCT; Lent et al., 1999) offered further articulation between career and academic development. SCCT asserts that preparedness for career transitions is a lifelong process that can be facilitated over the school years. The SCCT posits that, during the elementary and middle school years, children develop more realistic career self-efficacy and outcome expectations as well as construct provisional preferences and academic/occupational aspirations (Lent, 2004). These acquisitions highly rely on children's increased career exploration and social learning experiences, which can include teachers' feedback on the student's performance or one's perceived academic capabilities and psychophysiological reactions to the contents and tasks covered in academic and out-of-school settings. The SCCT is aligned with literature positing the need for children and youth to develop internal and external resources that help them cope with unstable environments (Di Fabio and Kenny, 2015). In addition, career and academic processes seem to jointly influence children's perseverance to overcome barriers and to attain goals, assignment of meaning to school and success in the later school-to-work transition (Bandura et al., 2001).

Empirical support to the complementary view of career and academic processes has been found. On the one hand, the academic experiences offer “a source of learning realistic occupational information” (Watson and McMahon, 2005, p. 124) and potentially afford the possibility for children to experience responsive, consistent and quality relationships with teachers as well as to engage in instrumental behaviors and advance career planning, exploration, aspirations, reasoning, agency, decidedness, cooperation and self-perceived employability (Howard et al., 2009, 2015; Veiga et al., 2014; Di Fabio and Kenny, 2015; Longobardi et al., 2016). On the other hand, career development advances students' learning self-regulation, construction of meaning about one-self, school engagement, achievement and satisfaction (Peetsma and van der Veen, 2011; Paradnikė and Bandzevičienė, 2016; Hartung, 2017; Rudolph et al., 2017).

Career and academic processes impact psychosocial outcomes later in life. Children's career exploration precedes career adaptability and identity later in adolescence and adulthood (Schmitt-Rodermund and Vondracek, 1999; Guan et al., 2015). Children's favorable career self-efficacy expectations promote career-choice readiness in adolescence (Hirschi, 2011). Evidence has additionally indicated that children's academic achievement is positively related with adult educational level and employment (Ek et al., 2005). Still, childhood literacy and numeracy learning difficulties have been shown to contribute, among other factors, to lower self-esteem, weak vocational identity and long-term unemployment (Anyadike-Danes and McVicar, 2005; Pasta et al., 2013; Chávez, 2016). School behavioral problems, weak academic achievement and learning disabilities during childhood seem also to increase the likelihood of school dropout and career indecision in adolescence and long-term unemployment in adulthood (Rojewski, 1996; Wiesner et al., 2003; Anyadike-Danes and McVicar, 2005; Ferreira et al., 2007). Still, factors such as the quality of the teacher-child relationship might protect students from such unfavorable trajectories (Pasta et al., 2013; Longobardi et al., 2016).

It is, therefore, important to theoretically and empirically acknowledge the linkages among career and academic processes to more deeply understand their mutual dynamics. The literature has suggested that career interventions implemented since the childhood period of the lifespan and systematically carried out over the school years positively affect students' academic achievement, school motivation, career exploration and awareness (Whiston et al., 2011; Choi et al., 2015). Career interventions may, therefore, benefit from theory and research that more deeply address the articulations among career and academic development.

Empirical findings to date have relied on longitudinal studies spanning middle school to adolescence or during the emerging adulthood period. Regarding the former, evidence suggested that a favorable future time perspective is associated with gains in academic achievement, which in turn has been shown to be a predictor and outcome of career planning (Peetsma and van der Veen, 2011; Negru-Subtirica and Pop, 2016). As for the latter, findings derived from emerging adults indicated that career self-efficacy expectations seem to be positively related with college persistence and academic achievement, which in turn impacts one's occupational choices by age 30 (Wright et al., 2012; Wang et al., 2015).

Prior research has also identified variations in career and academic processes across demographic characteristics. In general, girls demonstrate more favorable academic and career progress than boys (e.g., Bandura et al., 2001; Ferreira et al., 2007; Peetsma and van der Veen, 2011; Weis et al., 2013). The literature has also indicated that gender seems to moderate the child-in-context experiences and resulting career paths (Araújo and Taveira, 2009). On the other hand, the geographic location of children, although less addressed in the literature, has been shown to predict differences in life styles, income, occupational and employment opportunities (Rafecas, 2013), which in turn might contribute to differences in children's career preparedness and academic achievement (e.g., Howard et al., 2009). Previous exploratory studies in Portugal have also indicated that northern children are more motivated to solve social conflicts than their central peers (Pereira, 2014).

This study aims to further explore the linkages among career and academic development during the childhood period with a specific focus on articulations between career preparedness constructs (i.e., career self-efficacy, outcome expectations, exploration and planning) and academic achievement during middle school years. A four-wave longitudinal research design was employed. Gender and geographic location were tested as potential moderators affecting the articulation between career and school trends. Based on the reviewed literature and adopting a complementary position between career and academic processes, the following research hypotheses were tested:

Hypothesis 1: There will be positive associations between the career variables and academic achievement trends over time.
Hypothesis 2: Gender will moderate the association between the career and academic achievement trends over time.
Hypothesis 3: Geographic location will moderate the association between the career and academic achievement trends over time.

## Materials and methods

### Participants

Data from the four-wave longitudinal research project “Trajectories of childhood career development: A study with middle school children” (FCT-SFRH/BD/84162/2012) was employed to test the association between longitudinal trajectories of career and academic preparation. A non-probabilistic intentional sampling method was used to recruit participants aged 9 to 13 years old attending fifth-grade^1^ at the first occurrence of measurement through sixth-grade at the fourth occurrence of measurement. Portuguese fifth- and sixth-grades are aligned with level 2 of the International Standard Classification of Education (ISCED)—lower secondary education (OECD, European Union, UNESCO Institute for Statistics, 2015).

A total of four schools from northern and central Portugal collaborated in this project. Northern and central Portugal were defined according to a legal document approved by the European Union (i.e., Regulamento UE 868/2014)^2^. which organizes Portuguese regions from broader to more specific Nomenclatures of Territorial Units for Statistical Purposes. The initial sample included 446 children, 213 (47.8%) girls, and 233 (52.2%) boys, 314 (70.4%) from northern and 132 (29.6%) from central Portugal (*M*_ageWave1_ = 10.23, *SD* = 0.49). Attrition was found for 17 cases due to health problems, emigration, or failure to be promoted to the sixth-grade. Attrition was found to be missing at random, presenting no associations with demographic, career or academic variables. This study included participants with complete data across the four occurrences of measurement. The final sample included 429 children, 207 (48.3%) girls, and 222 (51.7%) boys, 298 (69.5%) from northern and 131 (30.5%) from central Portugal (*M*_ageWave1_ = 10.23, *SD* = 0.50). Most children were Portuguese-native (99.5%), except for two cases who were Chinese and Brazilian. Participants mostly derived from medium-low social economic status (52.4%), followed by low (28.2%) and medium-high statuses (18.6%)^3^.

### Measures

#### Career development

Children's self-efficacy expectations for academic, leisure and extracurricular activities were measured with the Portuguese version of the Multidimensional Scales of Perceived Self-Efficacy (MSPSE; Bandura, 1990; adapted by Teixeira and Carmo, 2004). Twenty-six items (e.g., “How easily do you plan your schoolwork”) were answered with a five-point Likert-type scale ranging from 1 “Not easy at all” to 5 “Very easy.” Higher scores reflected more favorable self-efficacy expectations for academic, leisure and extracurricular activities. Previous validity evidence of the Portuguese MSPSE version supported its hierarchical factor structure and theoretically predicted positive associations with academic achievement, career exploration and interests (e.g., Teixeira and Carmo, 2004; Oliveira, 2016). Reliability estimates in this sample ranged from 0.93 to 0.94 across the four occurrences of measurement.

Students' career exploratory outcome expectations were assessed with the Career Exploratory Outcome Expectations Scale (CEOES; Oliveira et al., 2016). This self-report measure includes 15 items (e.g., “Asking questions about the world of work at home, school and my friends will help me learn more about careers”) answered in a four-point Likert-type scale ranging from 1 “Very low or low probability” to 4 “Very high probability.” Higher scores reflected more favorable career exploratory outcome expectations. Extant validity results supported the CEOES one-dimensional structure, measurement invariance across both genders and positive association with career self-efficacy expectations (Oliveira et al., 2016). The estimates of internal consistency reliability in the current sample ranged from 0.80 to 0.91 over time.

Children's career exploration was assessed with the Childhood Career Exploration Inventory (CCEI; Oliveira, 2016). The CCEI is a self-report measure, which includes 12 items (e.g., “I like to ask my parents about their jobs”) answered in a five-point Likert-type scale ranging from 1 “Strongly disagree” to 5 “Strongly agree.” Higher scores indicated higher career exploration levels. Previous validity evidence supported the CCEI hierarchical factor structure, measurement invariance across genders during fifth- and sixth-grade as well as positive associations with self-concept and locus of control (Oliveira, 2016). Internal consistency reliability ranged from 0.79 to 0.86 in the current sample followed over time.

Career planning was measured with the Portuguese Childhood Career Development Scale version (CCDS; Schultheiss and Stead, 2004; adapted by Oliveira and Taveira, 2014). Eight items (e.g., “It is important for me to plan for a project”) were answered in a five-point Likert-type scale ranging from 1 “Strongly disagree” to 5 “Strongly agree.” Previous research with the Portuguese CCDS version suggested that career planning is positively associated with career exploration and locus of control (Oliveira and Taveira, 2014; Oliveira, 2016). Internal consistency reliability in this sample ranged from 0.88 to 0.91 across the four occurrences of measurement.

#### Academic achievement

Academic achievement was assessed through school records of the students' grades in each course at each occurrence of measurement. Average grades at each occurrence of measurement were computed. Higher average scores indicated higher school achievement.

### Ethical considerations and research procedures

This study followed Portuguese governmental regulatory standards to conduct research projects with children and youths at the school setting (Despacho, 15847/2007)^4^. This project was approved by the Portuguese Directorate General of Education, Ministry of Education and Science, through the monitoring system of school surveys (reference 0355100001). After obtaining such a legal approval, written consent to pursue the research project and recruit students from schools was obtained from institutional principals, teachers and school psychologists. The schools' principals and psychologists selected the classroom groups to which the project was introduced. All students from the classroom groups were invited to participate. Conforming with the Portuguese ethical standards of Psychology (Regulamento 258/2011)^5^, this study respected people's dignity and human rights, only including students who offered their assent for voluntary participation and whose caregivers signed a written consent form for their children's participation, acknowledging the research project's goals, procedures, confidentiality assurance and voluntary participation.

Data was collected from February 2013 to May 2014 by PhD and MSD Psychology students as well as school psychologists. Data at the first and second occurrences of measurement were collected 3 months apart. Six months elapsed between the second and third and between the third and fourth occasions. The MSPSE, CEOES, CCEI, and CCDS scales were group-administrated in the classroom setting. Researchers accessed school records to collect children's average grades at each occurrence of measurement. Taking the Portuguese ethical standards of Psychology and the longitudinal research demands into account, several strategies were employed to offer feedback to participants and to minimize attrition. These included offering make-up survey sessions for absent student, frequent school personal, e-mail and phone contacts, distribution of a newsletter informing caregivers of activities to advance their children's career development and academic achievement, as well as summaries of the main descriptive results from the study. Confidentiality was assured throughout this project.

### Data analyses

The Statistical Package for the Social Sciences (IBM SPSS, version 22.0 for Windows) was used to conduct the data analyses. A maximum 1.1% of missing values was found across the constructs at each occurrence of measurement. Results from the Little MCAR test suggested a completely at random pattern of missingness. An exception was only found for the planning items at the third occurrence of measurement. This exception was due to one case who did not complete the scale's items, whereby this participant's data was filtered from the analyses. The expectation-maximization method was used to treat the remaining missing values, due to their low frequency and random pattern (Tabachnick and Fidell, 2013).

Average and orthnormalized linear, quadratic and cubic trends of each career variable and school achievement (*x*) were computed, based on the following equations:

$$\begin{array}{l} Linear\;trend\;\left(x\right)=\frac{\begin{array}{c} \left(-3x_{{\left[Wave1\right]}^{\;}}\right)+\left(-1x_{{\left[Wave2\right]}^{\;}}\right)\;+\\ \left(1x_{{\left[Wave3\right]}^{\;}}\right)+\left(3x_{{\left[Wave4\right]}^{\;}}\right) \end{array}}{\sqrt{-3^{2}+\left({-1}^{2}\right)+1^{2}+3^{2}\;}}\\ Quadratic\;trend\;\left(x\right)=\frac{\begin{array}{c} \left(1x_{{\left[Wave1\right]}^{\;}}\right)+\left(-1x_{{\left[Wave2\right]}^{\;}}\right)\;+\\ \left(-1x_{{\left[Wave3\right]}^{\;}}\right)+\left(1x_{{\left[Wave4\right]}^{\;}}\right) \end{array}}{\sqrt{1^{2}+\left(-1^{2}\right)+\left(-1^{2}\right)+1^{2}}}\\ Cubic\;trend\;\left(x\right)=\frac{\begin{array}{c} \left(-1x_{{\left[Wave1\right]}^{\;}}\right)+\left(3x_{{\left[Wave2\right]}^{\;}}\right)\;+\\ \left(-3x_{{\left[Wave3\right]}^{\;}}\right)+\left(1x_{{\left[Wave4\right]}^{\;}}\right) \end{array}}{\sqrt{-1^{2}+3^{2}+\left({-3}^{2}\right)+1^{2}}} \end{array}$$

**Figure 1:**
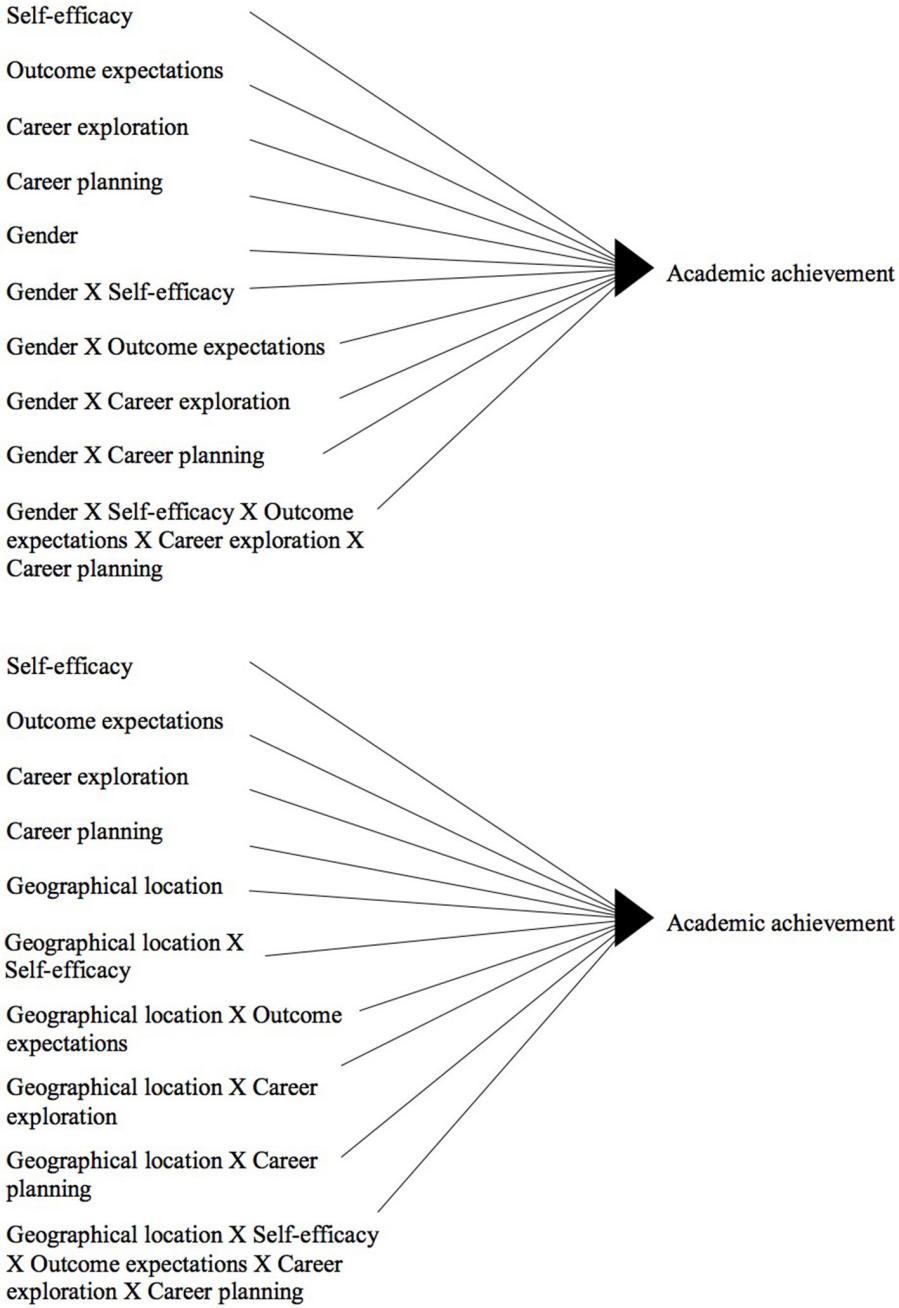
**Schematic representation of the regression models testing the gender and geographic area moderator effects on the linkage between career preparedness and school achievement**.

The associations between the career variables and academic achievement trends were tested. Due to evidence of non-normality of the sampling distribution, parametric (i.e., Pearson) and non-parametric (i.e., Spearman) correlation coefficients were computed. When both tests were consistent, parametric results were reported; otherwise, non-parametric results were presented (Fife-Schaw, 2006). Multiple simultaneous linear regression models were computed to test the predictive role of the career constructs on academic achievement trends. The Durbin-Watson statistics suggested that residuals were uncorrelated. Based on the Cook's Distance measure and the range of the standardized residuals, multivariate outliers were identified and filtered from the regression analyses to prevent their biases on the estimated models (Field, 2009). The gender and the geographic location moderator effects were investigated by adding interaction terms into the regression models of each trend (see Figure 1). Variables were centralized before computing the interaction terms (Aiken and West, 1991).

## Results

The correlational results suggested a positive and statistically significant association among children's career exploratory outcome expectations and academic achievement average trends, *r* = 0.28, *p* < 0.001. Overall, children who reported favorable career exploratory outcome expectation presented significantly high academic achievement. In turn, a negative and statistically significant correlation between career planning and academic achievement quadratic trends was found, *r* = −0.10, *p* = 0.04. Changes in career planning were inversely associated with changes in academic achievement. Career self-efficacy expectations and academic achievement quadratic trends also demonstrated a negative and statistically marginally significant association, *r* = −0.09, *p* = 0.07. Children's shifts from favorable to unfavorable self-efficacy expectations tended to be associated with shifts from lower to higher academic achievement (see Table 1).

**Table 1:** **Correlations between the career variables and academic achievement trends**.

**Variables**	**Self-efficacy**	**Outcome expectations**	**Exploration**	**Planning**	**Achievement**
**AVERAGE TREND**					
Self-efficacy	−				
Outcome expectations	0.07	−			
Exploration	0.40^***^	0.04	−		
Planning	0.47^***^	0.10^*^	0.55^***^	−	
Achievement	−0.05	0.28^***^	−0.08	−0.05	−
**LINEAR TREND**					
Self-efficacy	−				
Outcome expectations	0.004	−			
Exploration	0.28^***^	−0.05	−		
Planning	0.32^***^	−0.05	0.45^***^	−	
Achievement	−0.05	−0.01	0.08	0.02	−
**QUADRATIC TREND**					
Self-efficacy	−				
Outcome expectations	−0.08^†^	−			
Exploration	0.21^***^	0.04	−		
Planning	0.23^***^	−0.04	0.27^***^	−	
Achievement	−0.09^†^	0.08	−0.004	−0.10^*^	−
**CUBIC TREND**					
Self-efficacy	−				
Outcome expectations	0.01	−			
Exploration	0.20^***^	0.02	−		
Planning	0.23^***^	−0.02	0.22^***^	−	
Achievement	0.01	−0.04	−0.03	−0.001	−

* *p < 0.05*.

*** *p < 0.001*.

† *p < 0.10*.

The multiple linear regression model testing the predictive role of the career variables on academic achievement average trends explained 8.7% of the variance (*R*^2^ Adj. = 0.08) and was statistically significant, *F*_(4, 424)_ = 10.11, *p* < 0.001. Career exploratory outcome expectations constituted a statistically significant predictor of academic achievement average trends, β = 0.29, *t* = 6.12, *p* < 0.001. Children's overall favorable career exploratory outcome expectations were related with high academic achievement.

In addition, the linear regression model testing academic achievement regressed onto the career variables quadratic trends explained 2.7% of the variance (*R*^2^ Adj. = 0.02) and was statistically significant, *F*_(4, 417)_ = 2.86, *p* = 0.02. Career planning was a statistically significant predictor of academic achievement quadratic trends, β = −0.11, *t* = −2.23, *p* = 0.03. However, the constructs' progress trends were inversely related, as career planning gains and losses were significantly associated with losses and gains in academic achievement (see Table 2).

**Table 2:** **Multiple linear regressions testing academic achievement regressed onto career variables trends**.

**Predictors**	***R*^2^ (*R*^2^ Adj.)**	***F***	**β**	***t***
**AVERAGE TREND**				
Self-efficacy	0.09 (0.08)	10.11^***^	−0.03	−0.56
Outcome expectations			0.29	6.12^***^
Exploration			−0.06	−1.04
Planning			−0.04	−0.59
**LINEAR TREND**				
Self-efficacy	0.01 (0.004)	1.40	−0.05	−1.05
Outcome expectations			−0.004	−0.09
Exploration			0.12	2.20^*^
Planning			−0.01	−0.25
**QUADRATIC TREND**				
Self-efficacy	0.03 (0.02)	2.86^*^	−0.06	−1.24
Outcome expectations			0.08	1.66
Exploration			0.03	0.62
Planning			−0.11	−2.23^*^
**CUBIC TREND**				
Self-efficacy	0.008 (−0.002)	0.79	0.05	0.96
Outcome expectations			−0.05	−0.95
Exploration			−0.06	−1.26
Planning			−0.01	−0.24

* *p < 0.05*.

*** *p < 0.001*.

Tables 3, 4 summarize the results from the multiple linear regression models testing the gender and geographic location moderator effects in the linkages among career and academic trends.

**Table 3:** **Gender moderator role in the association among career variables and academic achievement trends**.

**Predictors**	***R*^2^ (*R*^2^ Adj.)**	***F***	**β**	***t***
**AVERAGE TREND**				
Self-efficacy	0.10 (0.08)	4.77^***^	−0.04	−0.69
Outcome expectations			0.30	5.90^***^
Exploration			−0.07	−1.27
Planning			−0.02	−0.38
Gender			−0.07	−1.41
Gender X self-efficacy			0.08	1.40
Gender × outcome expectations			−0.06	−1.19
Gender × exploration			−0.01	−0.25
Gender × planning			−0.06	−1.06
Gender × self-efficacy × outcome expectations × exploration × planning			0.06	1.22
**LINEAR TREND**				
Self-efficacy	0.03 (0.01)	1.46	−0.04	−0.84
Outcome expectations			−0.02	−0.32
Exploration			0.12	2.07^*^
Planning			−0.01	−0.15
Gender			−0.02	−0.31
Gender × self-efficacy			−0.01	−0.15
Gender × outcome expectations			−0.01	−0.26
Gender × exploration			0.08	1.49
Gender × Planning			−0.07	−1.20
Gender × self-efficacy × outcome expectations × exploration × planning			−0.12	−2.50^**^
**QUADRATIC TREND**				
Self-efficacy	-0.04 (0.01)	1.60	−0.07	−1.38
Outcome expectations			0.09	1.75^†^
Exploration			0.03	0.64
Planning			−0.10	−1.94^*^
Gender			0.002	0.04
Gender × self-efficacy			−0.04	−0.69
Gender × outcome expectations			0.01	0.19
Gender × exploration			0.002	0.03
Gender × planning			−0.09	−1.70
Gender × self-efficacy × outcome expectations × exploration × Planning			0.02	0.46
**CUBIC TREND**				
Self-efficacy	0.04 (0.01)	1.49	0.04	0.85
Outcome expectations			−0.02	−0.40
Exploration			−0.06	−1.12
Planning			−0.02	−0.35
Gender			−0.06	−1.24
Gender × self-efficacy			0.10	1.96^*^
Gender × outcome expectations			0.04	0.79
Gender × exploration			−0.003	−0.06
Gender × planning			−0.14	−2.66^**^
Gender × self-efficacy × outcome expectations × exploration × planning			−0.04	−0.77

† *p < 0.10*.

* *p < 0.05*.

** *p < 0.01*.

*** *p < 0.001*.

**Table 4:** **Geographic location moderator role in the association among career variables and academic achievement trends**.

**Predictors**	***R*^2^ (*R*^2^ Adj.)**	***F***	**β**	***t***
**AVERAGE TREND**				
Self-efficacy	0.18 (0.16)	9.21^***^	−0.08	−1.51
Outcome expectations			0.30	7.91^***^
Exploration			0.01	0.25
Planning			0.02	0.36
Geographic location			0.16	2.86^**^
Geo × self-efficacy			0.04	0.83
Geo × outcome expectations			−0.29	−6.14^***^
Geo × exploration			−0.04	−0.77
Geo × planning			0.05	0.79
Geo × self-efficacy × outcome expectations × exploration × planning			−0.03	−0.52
**LINEAR TREND**				
Self-efficacy	0.02 (−0.001)	0.94	−0.06	−1.19
Outcome expectations			−0.008	−0.17
Exploration			0.13	2.34^*^
Planning			−0.02	−0.35
Geographic location			−0.04	−0.77
Geo × self-efficacy			−0.07	−1.42
Geo × outcome expectations			0.01	0.14
Geo × exploration			−0.04	−0.64
Geo × planning			0.03	0.50
Geo × self-efficacy × outcome expectations × exploration × planning			−0.02	−0.35
**QUADRATIC TREND**				
Self-efficacy	0.07 (0.04)	2.90^**^	−0.05	−1.09
Outcome expectations			0.06	1.26
Exploration			0.04	0.71
Planning			−0.05	−0.90
Geographic location			−0.16	−3.06^**^
Geo × self-efficacy			0.07	1.33
Geo × outcome expectations			0.03	0.52
Geo × exploration			0.00	−0.008
Geo × planning			−0.08	−1.50
Geo × self-efficacy × outcome expectations × exploration × planning			0.09	1.72^†^
**CUBIC TREND**				
Self-efficacy	0.02 (−0.01)	0.69	0.01	0.09
Outcome expectations			−0.05	−0.93
Exploration			−0.03	−0.63
Planning			0.01	0.15
Geographic location			−0.06	−1.15
Geo × self-efficacy			−0.06	−1.09
Geo × outcome expectations			0.01	13
Geo × exploration			0.06	1.14
Geo × planning			07	1.25
Geo × self-efficacy × Outcome expectations × exploration × planning			−0.05	−0.96

† *p < 0.10*.

* *p < 0.05*.

** *p < 0.01*.

*** *p < 0.001*.

**Figure 2:**
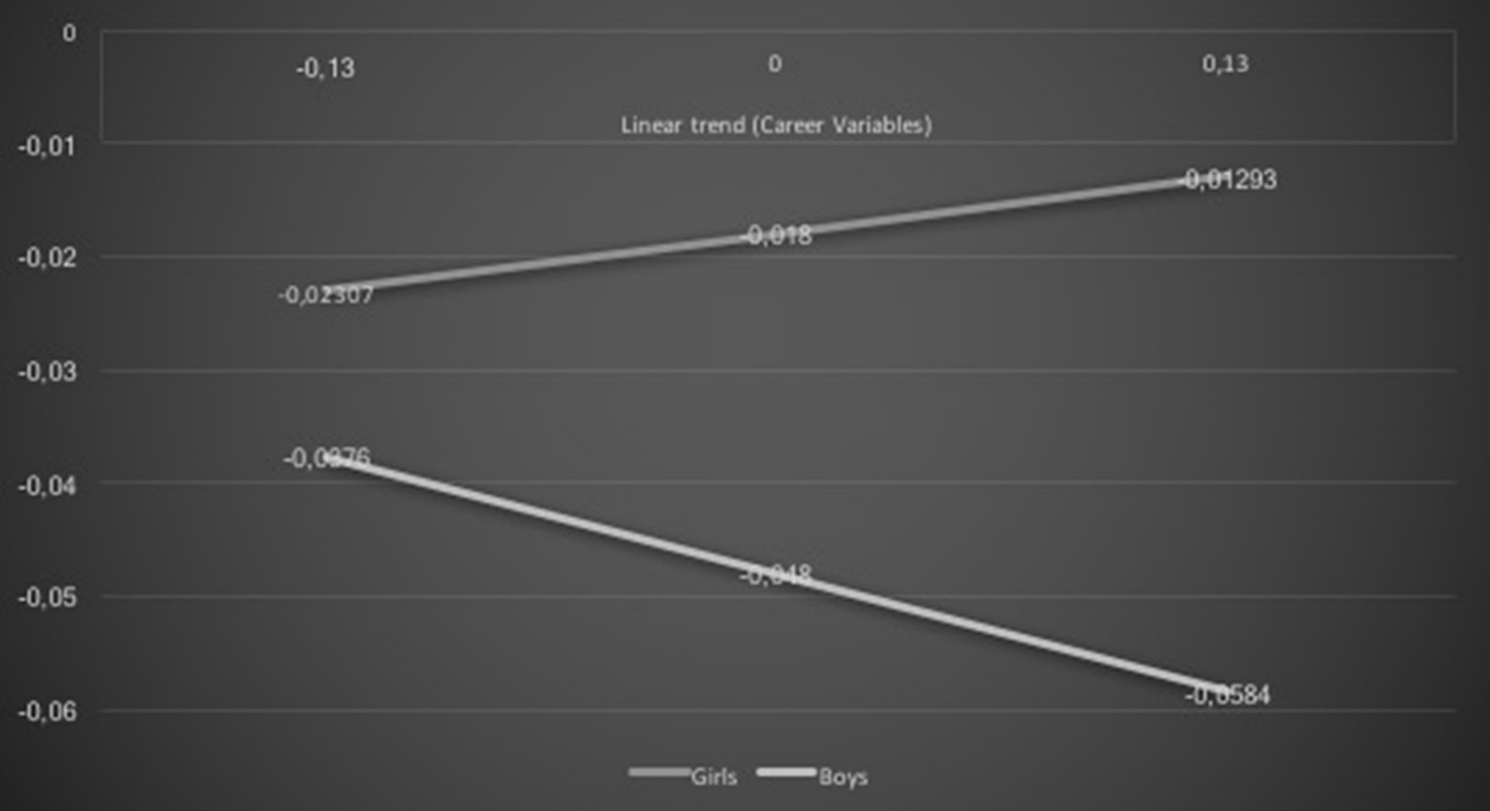
**Gender and career variables interaction effect on academic achievement: linear trends**.

Results suggested that gender was a statistically significant moderator of the association between career variables and academic achievement linear trends, β = −0.12, *t* = −2.50, *p* = 0.01. While girls presented a positive association among the constructs, boys presented a negative association (see Figure 2).

**Figure 3:**
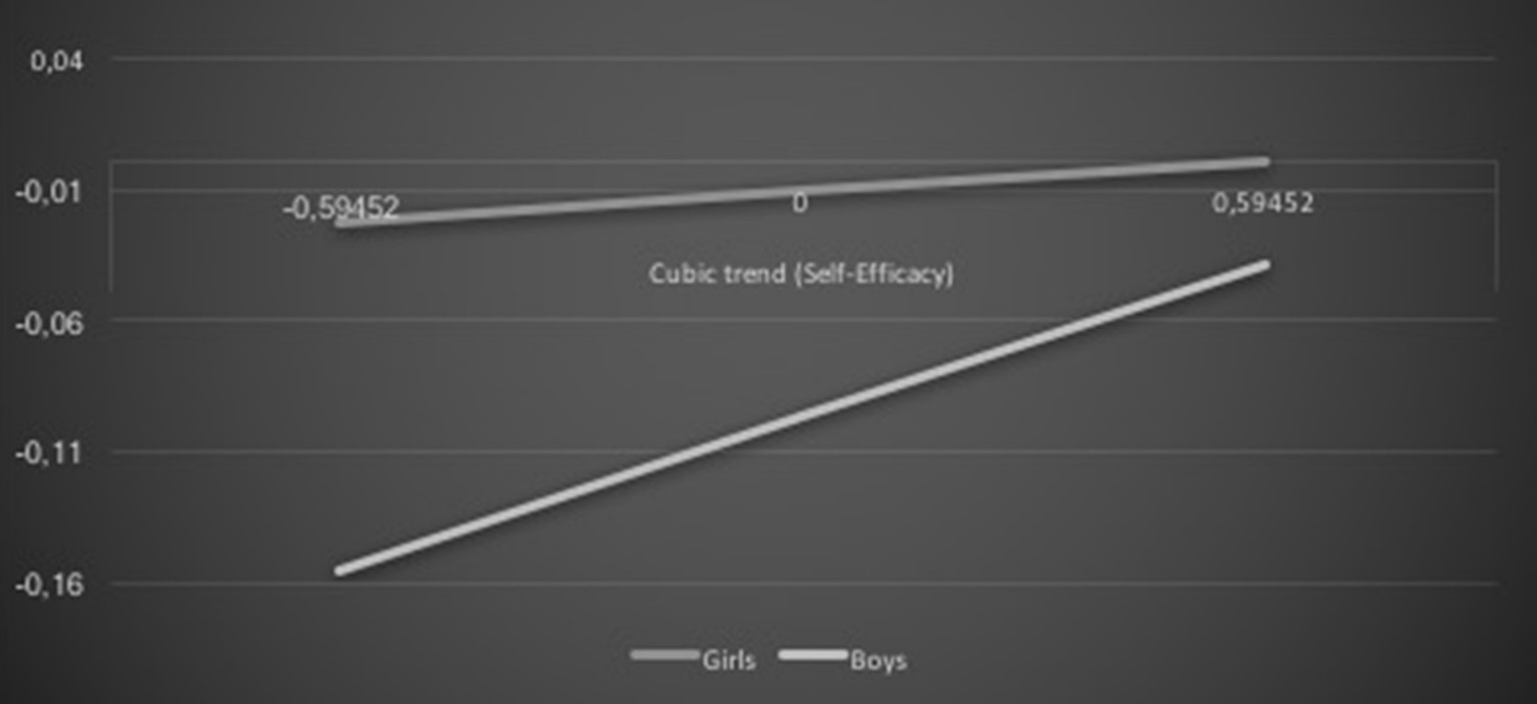
**Gender and self-efficacy interaction effect on academic achievement: cubic trends**.

**Figure 4:**
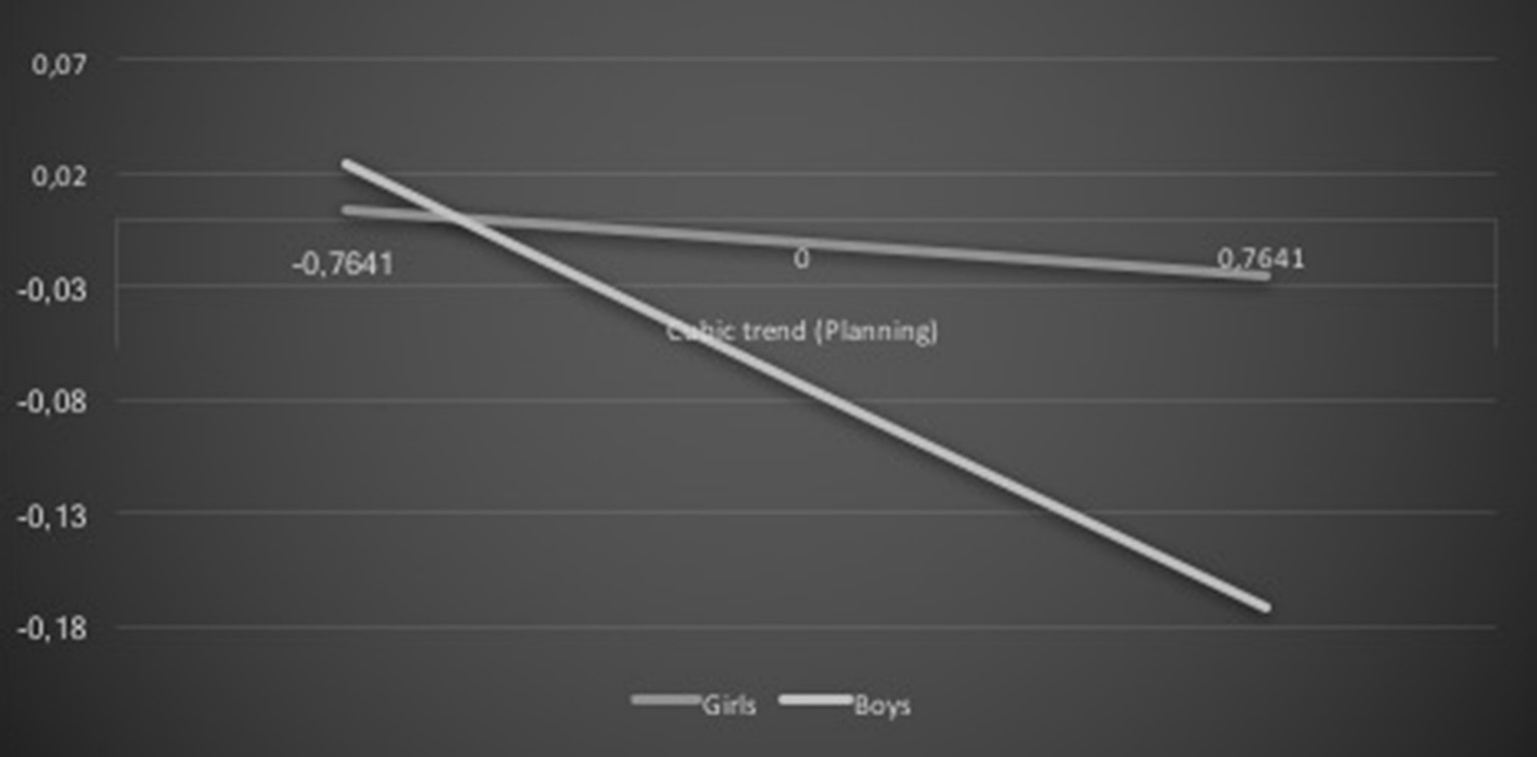
**Gender and career planning interaction effect on academic achievement: cubic trends**.

Gender also moderated the association between career self-efficacy and academic achievement, β = 0.10, *t* = 1.96, *p* = 0.05, as well as among career planning and academic achievement cubic trends, β = −0.14, *t* = −2.66, *p* = 0.008. The positive association was more pronounced for boys than girls (see Figure 3). In addition, the career planning cubic trend was negatively associated with the academic achievement cubic trend for both genders, but was stronger for boys than girls (see Figure 4).

**Figure 5:**
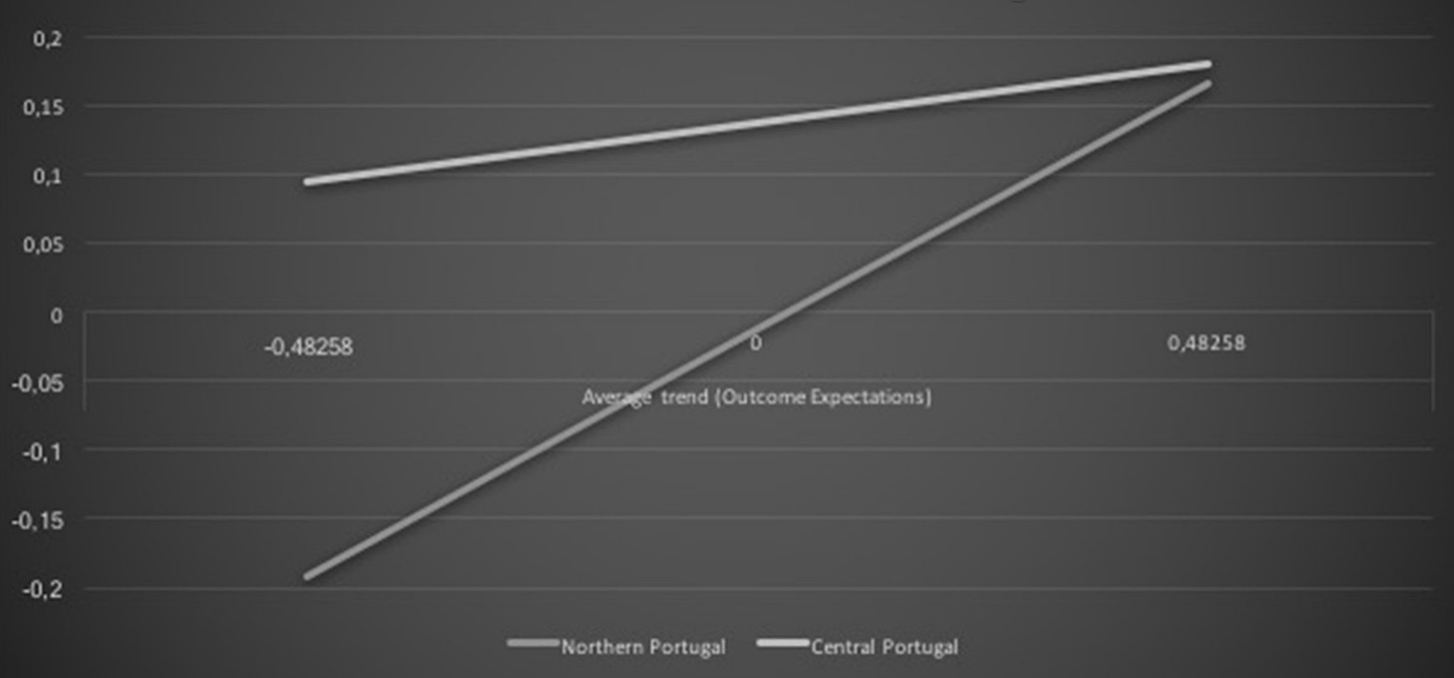
**Geographic location and outcome expectations interaction effect on academic achievement: average trends**.

Additionally, geographic location of the children moderated the association between career exploratory outcome expectations and academic achievement average trends, β = −0.29, *t* = −6.14, *p* < 0.001. Children from both geographical areas presented a positive association among the overall career exploratory outcome expectations and academic achievement trends. More favorable career exploratory outcome expectations were associated with higher academic achievement over time. However, this trend was more pronounced for northern Portuguese children than for their central Portuguese peers (see Figure 5).

**Figure 6:**
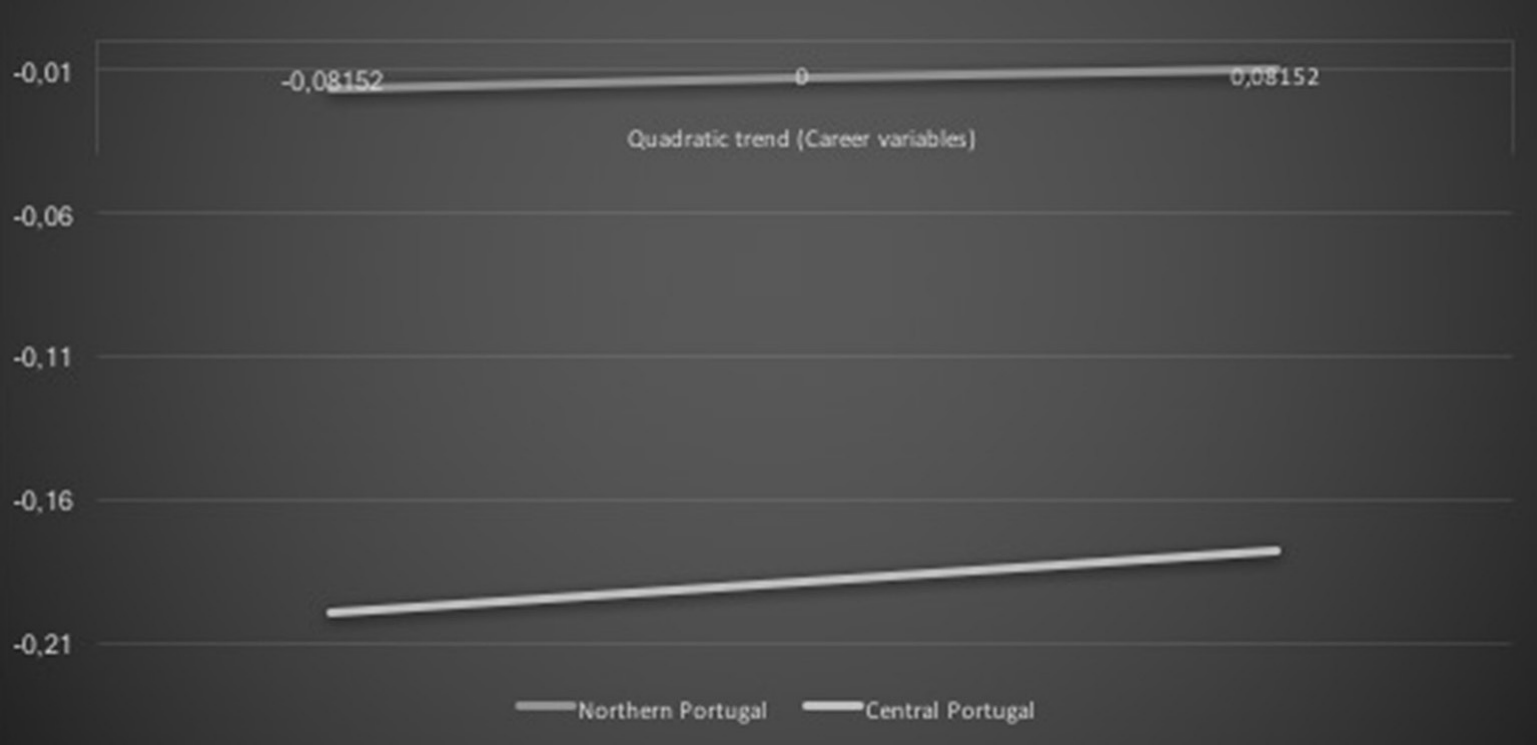
**Geographic location career variables interaction effect on academic achievement: quadratic trends**.

The relationships between the career variables and academic achievement quadratic trends were positively related and moderated by the geographic location, β = 0.09, *t* = 1.71, *p* = 0.09. The positive association tended to be slightly stronger for central Portuguese children than northern Portuguese children (see Figure 6).

## Discussion

This study examined longitudinal trends between career preparedness constructs and academic achievement with Portuguese middle school children. The results support the need to acknowledge the joint linkages among career and academic dynamics, as several statistically significant relations and predictive models among the constructs were found.

H1 was partially supported. A positive association between career exploratory outcome expectations and academic achievement average trends was suggested by both correlational and regression results. The association among these constructs is aligned with literature suggesting that during childhood, students are expected to assign meaning to school and acknowledge the importance of academic achievement for their future careers (Super, 1994; Hartung, 2017). As children engage in instrumental behaviors and adapt to school social expectations (Chávez, 2016), they might set the goal of attaining good grades. This goal might foster a virtuous cycle of children's academic success and enhance their academic confidence in the future. The combination of positive performance and confidence may foster more active/meaningful learning, as well as the development of a sense of industry and establishment of a close teacher-student relationship (Erikson, 1963; Lent et al., 1999; Lent, 2004; Longobardi et al., 2016; Oliveira et al., 2016). On the contrary, accumulated experiences of school failure might foster a vicious cycle of future failure and diminished academic confidence, thus increasing the likelihood to avoid school-related tasks and to develop a sense of inferiority if no protective psychoeducational strategies are set in motion (Erikson, 1963; Anyadike-Danes and McVicar, 2005; Ferreira et al., 2007). Future research could examine how children's academic achievement might sustain and/or be sustained by optimism and favorable expectations to attain valued outcomes from career exploration.

Contrary to our hypothesis, academic achievement presented negative correlations with career self-efficacy and career planning quadratic trends, with the latter being similarly verified in regression analysis. These findings are surprising considering extant evidence pointing to a positive association among the constructs (e.g., Peetsma and van der Veen, 2011; Wright et al., 2012; Negru-Subtirica and Pop, 2016). The negative association between career self-efficacy expectations and academic achievement quadratic trends suggest that a high academic achievement peak was linked to an unfavorable self-efficacy valley. It is worth emphasizing that the MSPSE offers a global estimate of self-efficacy for academic, leisure and extracurricular activities, which might have influenced these findings. Despite temporal variations in academic achievement, a child's attainment of high academic achievement might foster stronger engagement in academic pursuits, which in turn, might constrain his/her availability to engage in extracurricular and leisure activities. Oppositely, a child's weak academic achievement might source from reduced engagement in academic activities and more engagement in extracurricular and leisure activities, thus increasing one's confidence in out-of-school activities and possibly inflating those self-efficacy scores. To clarify these findings, future research could address the importance of extracurricular and leisure activities in children's career and academic progress trends and how these various activities might mutually influence each other (Super, 1994; Araújo and Taveira, 2009; Hartung, 2017).

By a similar token, as students' academic achievement gains might spring from a great amount of time and effort devoted to academic activities, which might diminish the time available to consider their future. In turn, children demonstrating poorer academic achievement, might invest less time in academic pursuits and more time engaging in other pursuits (Veiga et al., 2014). It might also be the case that as children perform the student role (Super, 1994; Araújo and Taveira, 2009), their planning becomes more focused on academic tasks and management of student responsibilities than on planning for future roles. Hence, future studies employing both career and academic planning measures could help clarify this result. In addition, future studies could rely on mixed-method research designs to bring complementary qualitative evidence on the students' career-related reasoning (Howard et al., 2015; Watson et al., 2015) and examine its impact on the career planning-academic achievement association.

Both the correlational and regression analyses found no association between career exploration and academic achievement trends, which was misaligned with our initial hypothesis. Still, this finding seems consistent with literature acknowledging that the school setting may not sufficiently stimulate children's inquisitive behavior, reflexivity and career exploration (Kuijpers and Meijers, 2012; Negru-Subtirica and Pop, 2016). We, therefore, highlight extant calls to investigate career exploration in extracurricular and out-of-school activities as well as its associations with academic processes, such as engagement in school or academic adjustment (e.g., Araújo and Taveira, 2009; Oliveira, 2016; Paradnikė and Bandzevičienė, 2016). Moreover, future studies could assess academic achievement as perceived by the child rather than his/her recorded grades at the school setting. In previous studies, children perceived achievement has been shown to play a greater role in career and academic development than their actual grades (e.g., Bandura et al., 2001).

Taking the demographic characteristics into account, H2 was supported. Gender moderated the relationships between the career and academic achievement linear trends, with girls demonstrating a positive relationship and boys demonstrating the reverse. This finding is consistent with previous evidence suggesting that girls demonstrate more advanced career development and academic achievement than boys (e.g., Bandura et al., 2001; Ferreira et al., 2007; Weis et al., 2013). However, this study brings complementary longitudinal evidence to this topic by suggesting that boys' academic success might come with a cost of less advanced career development. This might suggest that, during middle school years, boys may not yet comprehend the role of school and academic achievement for their future, contrary to what would be expected from the literature (Super, 1980; Bandura, 1990; Peetsma and van der Veen, 2011). This possible explanation may similarly be applied to the results indicating opposite cubic trends between career planning and academic achievement, with such a relationship being more pronounced for boys than girls. Children's greater focus on academic demands may deviate their attention from future career prospects. This might be more evident for boys, as they seem to not yet articulate linkages between career and academic processes. Further studies employing mixed-method designs could clarify these results by addressing girls' and boys' discourse when talking about their role as a student and its links to the future.

Results also indicated similar associations among self-efficacy expectations and academic achievement cubic trends, although such a relation was more pronounced for boys compared to girls. This finding is aligned with literature highlighting the importance of self-efficacy expectations for academic achievement in childhood (Lent, 2004). The more pronounced results for boys compared to girls might suggest that boys' overall confidence in academic, extracurricular and leisure activities is central to their favorable academic achievement. Boys' academic success might, therefore, be facilitated when one acknowledges their multiple life roles, offers opportunities to learn how to manage time and enjoy both school and out-of-school activities, and recognizes their achievements in academic, extracurricular and leisure tasks. Although this seems also to be the case for girls, perhaps their confidence in leisure and extracurricular activities might be reduced and masked by the general self-efficacy measure used in this study. Future research should clarify the importance of extracurricular and leisure activities for children's career development and academic processes, taking differences for gender into consideration (Bandura et al., 2001).

H3 was also supported. Despite both geographic areas presenting a positive association among career exploratory outcome expectations and academic achievement average trends, such an association was more evident for northern than central Portugal. This finding might reflect the Portuguese social-economic regional asymmetries and the fact that the northern area typically offers increased educational and work opportunities than the central area (Rafecas, 2013). On the one hand, northern Portuguese children may benefit from greater opportunities to explore academic domains and related jobs, thus promoting academic achievement because of the more favorable career outcomes available in this region (Oliveira et al., 2016). On the other hand, the academic achievement of central Portuguese children seems not to be as facilitated by career exploratory outcome expectations. This might be due the economic disadvantageous situation of the central area (Rafecas, 2013), which might embolden children' efforts to be successful at school to increase the likelihood of future career attainment within a more difficult labor market. This possibility seems aligned with the additional result suggesting that central Portuguese children presented a slightly stronger positive association between the career constructs and academic achievement quadratic trends than their northern peers. The latter finding might suggest that central Portuguese children may articulate career and academic processes and eventually engage in academic activities as a strategy to succeed in the future more so than their northern peers do.

The results regarding the geographic moderator effects are, therefore, consistent with international evidence suggesting variations in career and academic variables for children's geographic areas (Howard et al., 2009). They are also aligned with calls to acknowledge contextual influences on a person's career development and school attainment (Lent et al., 1999; Lent, 2004). Future research could clarify the similarities and differences between northern and central Portuguese schools, acknowledging for example their learning environment, quality of teacher-student relationships and leadership features. Such a detailed description of northern and central Portuguese schools would afford the possibility to examine their impact on students' career and academic development, thus enlightening our findings.

This study relied on a sample with an unbalanced distribution of children from northern and central Portugal. Hence, caution is advised when generalizing the geographic location moderator results. To overcome such a limitation, future studies could be conducted with a larger sample of Portuguese middle school children and include a balanced number of participants per geographic location. Moreover, the geographic location could be expanded in future studies, recruiting children from the continent and isles of Portugal (Regulamento UE 868/2014)^6^ and examining the career and academic particularities of children from rural, urban and semi-urban settings. Following a multidisciplinary and collaborative approach (Watson et al., 2015), it would be important for career development researchers to establish partnerships with colleagues in the education, economy and sociology domains to examine the impact of the Portuguese regional asymmetries in children's career and academic processes as well as to jointly seek strategies to reduce such inequalities. It could also be relevant to conduct cross-cultural studies examining children's career and academic development trends in different countries. For example, research partnerships could be strengthened with other Portuguese-speaking countries in America (Brazil), Asia (Macau, East Timor), and Africa (Angola, Cape Verde, and Mozambique).

In conclusion, this study advanced an emerging body of literature assessing the linkages among career and academic processes (Watson et al., 2015; Negru-Subtirica and Pop, 2016). Relying on a longitudinal research design, this study highlighted the articulation between children's career preparedness and academic achievement, thus calling for additional efforts to address the linkages among these processes throughout the lifespan. This work might stimulate future research and program interventions. Regarding research, the complementary view of career and academic processes (Kuijpers and Meijers, 2012) grounding this study might trigger future multidisciplinary research efforts (Watson et al., 2015). Such efforts would be useful to deepen the scientific knowledge of career and academic processes and to continue investigating their mutual dynamics with longitudinal designs (Negru-Subtirica and Pop, 2016). Moreover, multidisciplinary research could identify common internal and external resources that facilitate students' positive development and psychosocial functioning (Erikson, 1963; Gottfredson, 1981; Di Fabio and Kenny, 2015; Chávez, 2016). Research comparing the career and academic processes of children with and without records of school behavioral problems or diagnosed learning disabilities could also be useful to discern their career and academic needs and to inform psychoeducational practices (Rojewski, 1996; Pasta et al., 2013). As for intervention, this study affirms the need for career practitioners to recognize the importance of the student role (Super, 1994) during the childhood period. This study affirms the potential value of jointly fostering career preparedness and academic achievement during the childhood period of the lifespan. To enhance career preparedness and academic achievement, career interventions could (a) help students identify and manage life roles; (b) create opportunities for students to observe behaviors and activities from their peers and adult practitioners, (c) stimulate mastery and performance experiences in school and out-of-school settings, and (d) help students identify and activate their social support networks (e.g., Bandura et al., 2001; Lent, 2004; Howard et al., 2009; Di Fabio and Kenny, 2015; Oliveira et al., 2016; Paradnikė and Bandzevičienė, 2016). Taking the importance of demographic factors into account, career practitioners could also develop interventions with educational agents (e.g., teachers, parents) to acknowledge the impact of their quality relationships, and affirm their vital role in transmitting gender and prestige balanced messages (Gottfredson, 1981; Pasta et al., 2013; Longobardi et al., 2016). These educational efforts could also encourage further collaboration between teachers, parents, and the workforce to facilitate children's overall positive career and academic development (Araújo and Taveira, 2009; Watson et al., 2015).

## Ethics statement

This study was carried out in accordance with the Portuguese Ethical Standards of Psychology and the Portuguese Directorate General of Education. All participants’ caregivers gave written informed consent in accordance with the Declaration of Helsinki. The data collection protocol was approved by the Portuguese Monitoring System of school surveys.

## Author contributions

IO Contributed to the conception and draft of the manuscript. MT and EP Contributed for its design and critical review. All authors revised and approved the manuscript's version to be submitted and assured the integrity and accuracy of the entire work.

## Funding

This study was funded by the Portuguese Foundation for Science and Technology through a Doctoral grant (SFRH/BD/84162/2012), supported by national funds of the Portuguese Ministry of Education and Science and the European Social Fund through the Human Capital Operational Program. This study was conducted at the Psychology Research Centre (UID/PSI/01662/2013), University of Minho, which is supported by the Portuguese Foundation for Science and Technology and the Portuguese Ministry of Education and Science through national funds and co-financed by FEDER through COMPETE2020 under the PT2020 Partnership Agreement (POCI-01-0145-FEDER-007653).

### Conflict of interest statement

The authors declare that the research was conducted in the absence of any commercial or financial relationships that could be construed as a potential conflict of interest.

## Footnotes

^1^The Portuguese educational system includes four compulsory cycles of study. The first cycle of study (i.e., ISCED level 1 primary education) ranges from the first- to the fourth-grade and usually includes children aged six to 10. The second cycle of study includes the fifth- and sixth-grade and is usually attended by children aged 10 to 11. The third cycle of study comprises seventh-, eighth- and ninth-grade, usually including students aged 11 to 14. The Portuguese second and third cycles of study both correspond to the ISCED level 2 lower secondary education. The fourth cycle of study (i.e., ISCED level 3 upper secondary education) spins from tenth- through twelfth-grade and is usually attended by students aged 14 to 17.

^2^Regulamento UE n.° 868 da Comissão (2014).

^3^Social economic statuses relied on the caregivers' educational level and occupation, following the Portuguese Classification of Occupations (Instituto Nacional de Estatística, 2011). A low social economic status was coded when caregivers did not complete Portuguese compulsory education and held unqualified jobs. A medium-low status was coded when at least one caregiver completed the Portuguese compulsory education and held a semi-qualified job. A medium-high status was coded when at least one of the caregivers had a college degree and held a qualified job.

^4^Despacho Ministério da Educação Diário da República Portuguesa n.° 15847 (2007).

^5^Regulamento Portuguese Order of Psychologists n.° 258 (2011).

^6^Regulamento UE n.° 868 da Comissão (2014).
